# When the placebo effect is not an effect

**DOI:** 10.1080/17453674.2021.1969155

**Published:** 2021-08-25

**Authors:** Ian Harris

**Affiliations:** Ingham Institute for Applied Medical Research, South Western Sydney Clinical School, UNSW Sydney, Australia

In this issue of the journal, Rosén et al. report the results of a survey of surgeons regarding placebo effects.^1^ In particular, they show that surgeons consider “non-specific” effects (including aspects of the surgeon-patient interaction) and “placebo effects” to be important. Their definitions of these terms are clearly stated but not universally accepted. A discussion of these terms is necessary to allow surgeons to better understand the reasons *why* people improve after surgery.

The terms placebo effect, non-specific effects and contextual effects all refer to responses that are separate to the specific effects of surgery: the effect that results from the anatomical and physiological changes brought about by the surgical procedure. The specific surgical effect is best measured in a high-fidelity placebo trial in which all things are equal *except* the specific part of the procedure in question.^2^ If the surgical group has better outcomes than the placebo group, it can be implied that the difference between the groups was due entirely to the specific effect of the surgery.

However, in many placebo surgical trials in orthopaedics, the difference in outcome between placebo surgery and the surgical procedure is not significant, i.e., there is no specific effect of surgery.^3–7^ Importantly, however, is the observation that in all of these studies, both groups (surgery and placebo surgery) show significant improvements after surgery. What is of interest, and what needs defining, is what causes the observed improvement when that improvement is not due to the specific effect of surgery.

Using the term “placebo effect” to describe the improvement seen after a placebo procedure is common. It is the definition used by Rosén et al. ^1^ and the definition used in my own book.^8^ The problem with using that definition is that it suggests that the improvement seen after placebo surgery is *due to* the placebo. In reality, the improvement is likely to have occurred without the placebo surgery. In fact, placebos, by definition, have no direct effect themselves.

Some of the improvement seen after placebo surgery may be due to contextual effects; what Rosén et al refer to as non-specific effects and others have referred to as “ritual” effects.^9^ These include patient expectations, the confidence and personality of the surgeon, and even the cost of the procedure. While contextual effects may explain some of the improvement after surgery, these effects are often short-lived and are unlikely to explain larger, sustained improvements.

Three other, often overlooked factors are likely to explain most or all of the improvement after surgery seen in placebo surgical trials, and in much of the surgery we perform in clinical practice. These factors are natural history, regression to the mean and concomitant treatment.

Natural history (what would happen regardless of treatment) explains, for example, the eventual resolution of symptoms from the common cold. It also explains most or all of the improvement in pain that occurs after injuries. Natural history also confounds the treatment of fluctuating conditions such as multiple sclerosis. Without considering natural history in trials of surgery, we may falsely attribute improvement that occurs post-surgery as being due to the surgery (the fallacy of *post hoc ergo propter hoc*: it follows, therefore it is because of).

Regression to the mean occurs when we select patients who are currently at one end of a spectrum and follow them. Over time, they will fall closer to the mean. A good example is provided by Daniel Kahneman in his book, Thinking, Fast and Slow. He described an experienced flight instructor who recommended punishing bad performance (those at the lower end of the spectrum) and not praising good performance (those at the high end of the spectrum), saying: “On many occasions I have praised well performing cadets… the next time they usually do worse. On the other hand, I have often screamed into a cadet’s earphone for bad execution, and in general he does better on his next try”.^10^ Both groups were simply regressing to the mean.

Similarly, only selecting people with severe knee pain from a pool of people with osteoarthritis of the knee (a condition in which symptoms fluctuate widely) will make any treatment look good. The average pain in that group will fall closer to the mean over time and, similarly, others in the pool who were not selected will have severe pain later. Regression to the mean is likely to explain the similar improvement reported in knee arthritis symptoms for the multitude of therapies in which a before-and-after analysis is performed.

The phenomena of natural history and regression to the mean are strong reasons for a control group in any study and why caution is advised when interpreting non-comparative studies.

**Figure:**
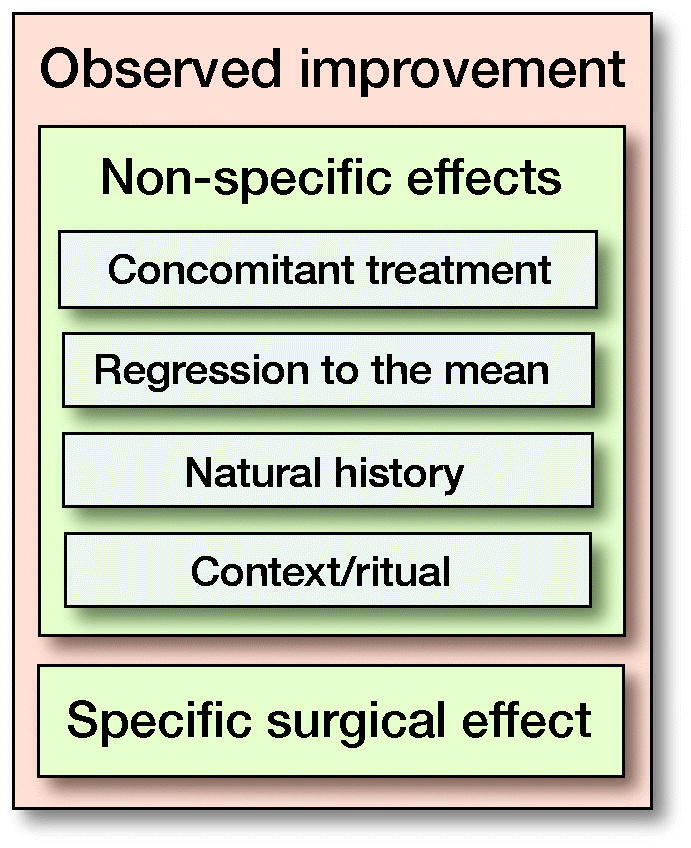


Concomitant treatment is also often overlooked. In a landmark trial comparing (the then new) bone morphogenetic protein (BMP) to (traditional) bone grafting in the treatment of ununited tibia fractures, BMP (with a union rate of 75%) was deemed equally effective as bone grafting (with a union rate of 84%). However, all patients also received intramedullary nailing, which produces similar union rates when used *alone* as a treatment for ununited tibia fractures.^11^ It is quite possible that BMP (and bone grafting) added nothing, and that the perceived effectiveness was entirely due to the concomitant treatment of intramedullary nailing.

Placebo surgical trials have a great advantage in determining the effectiveness of surgery because they provide the same contextual effects in both groups, patients are blinded and all other factors are equal between groups. It is my opinion, however, that the use of the term “placebo effect” to describe all the improvement seen after surgery has led to the widespread misinterpretation that the placebo treatment causes the improvement. Surgeons need to understand all the factors that contribute to improvements seen after surgery. These include, apart from any specific effects of surgery, contextual effects around the procedure, the effect of concomitant treatments, and what would have happened anyway (natural history and regression to the mean), and are summarized in the Figure.

Surgeons should also understand that components of the Figure are not to scale. In other words, the relative role of each component will vary widely depending on the procedure. For example, in the surgical management of proximal humerus fractures, it is likely that the specific surgical effect is small, there may be some contextual effects, and the effects of natural history and concomitant treatment (post-operative physical therapy) may be strong. Conversely, the specific surgical effect of surgery to stabilize an unstable knee may be very large, and the role of other effects minor. While the definitions of some or all these effects vary, it is important for surgeons to understand the factors contributing to the observed improvement after surgery.


Ian Harris *Ingham Institute for Applied Medical Research, South Western Sydney Clinical School, UNSW Sydney, Australia*
*Email:*
iaharris1@gmail.com


